# Exposure to a 900 MHz electromagnetic field induces a response of the honey bee organism on the level of enzyme activity and the expression of stress-related genes

**DOI:** 10.1371/journal.pone.0285522

**Published:** 2023-05-12

**Authors:** Pawel Migdal, Paweł Bieńkowski, Małgorzata Cebrat, Ewelina Berbeć, Mateusz Plotnik, Agnieszka Murawska, Przemysław Sobkiewicz, Agnieszka Łaszkiewicz, Krzysztof Latarowski

**Affiliations:** 1 Department of Environment, Hygiene and Animal Welfare, Bee Division, Wroclaw University of Environmental and Life Sciences, Wroclaw, Poland; 2 Telecommunications and Teleinformatics Department, Wroclaw University of Science and Technology, Wroclaw, Poland; 3 Laboratory of Molecular and Cellular Immunology, Hirszfeld Institute of Immunology and Experimental Therapy, Polish Academy of Sciences, Wroclaw, Poland; 4 Department of Human Nutrition, Wroclaw University of Environmental and Life Science, Wroclaw, Poland; King Saud University, SAUDI ARABIA

## Abstract

There are many artificial sources of radiofrequency electromagnetic field (RF-EMF) in the environment, with a value between 100 MHz and 6 GHz. The most frequently used signal is with a frequency of around 900 MHz. The direction of these changes positively impacts the quality of life, enabling easy communication from almost anywhere in the world. All living organisms in the world feel the effects of the electromagnetic field on them. The observations regarding the influence of a RF-EMF on honey bees, describing the general impact of RF-EMF on the colony and/or behavior of individual bees, such as reduction in the number of individuals in colonies, extended homing flight duration, decrease in breeding efficiency, changes in flight direction (movement of bees toward the areas affected by RF-EMF), increase in the intensity and frequency of sounds characteristic for those announcing the impending danger. In this work, we describe the changes in the levels of some of the stress-related markers in honey bees exposed to varying intensities and duration of RF-EMF. One-day-old honeybee worker bees were used for the study. The bees were randomly assigned to 9 experimental groups which were exposed to the following 900 MHz EMF intensities: 12 V/m, 28 V/m, and 61 V/m for 15 min, 1 h and 3 h. The control group was not exposed to the RF-EMF. Each experimental group consisted of 10 cages in which were 100 bees. Then, hemolymph was collected from the bees, in which the activity was assessed AST, ALT, ALP, GGTP, and level of nonenzymatic antioxidants albumin, creatinine, uric acid, and urea. Bees were also collected for the analysis of rps5, ppo, hsp10, hsp70, hsp90, and vitellogenin gene expression. Our study shows that exposure to a 900 MHz electromagnetic field induces a response in the honey bees that can be detected in the level of enzyme activity and the expression of stress-related genes. The response is similar to the one previously described as a result of exposition to UVB irradiation and most likely cannot be attributed to increased temperature.

## Introduction

**Fig 1:**
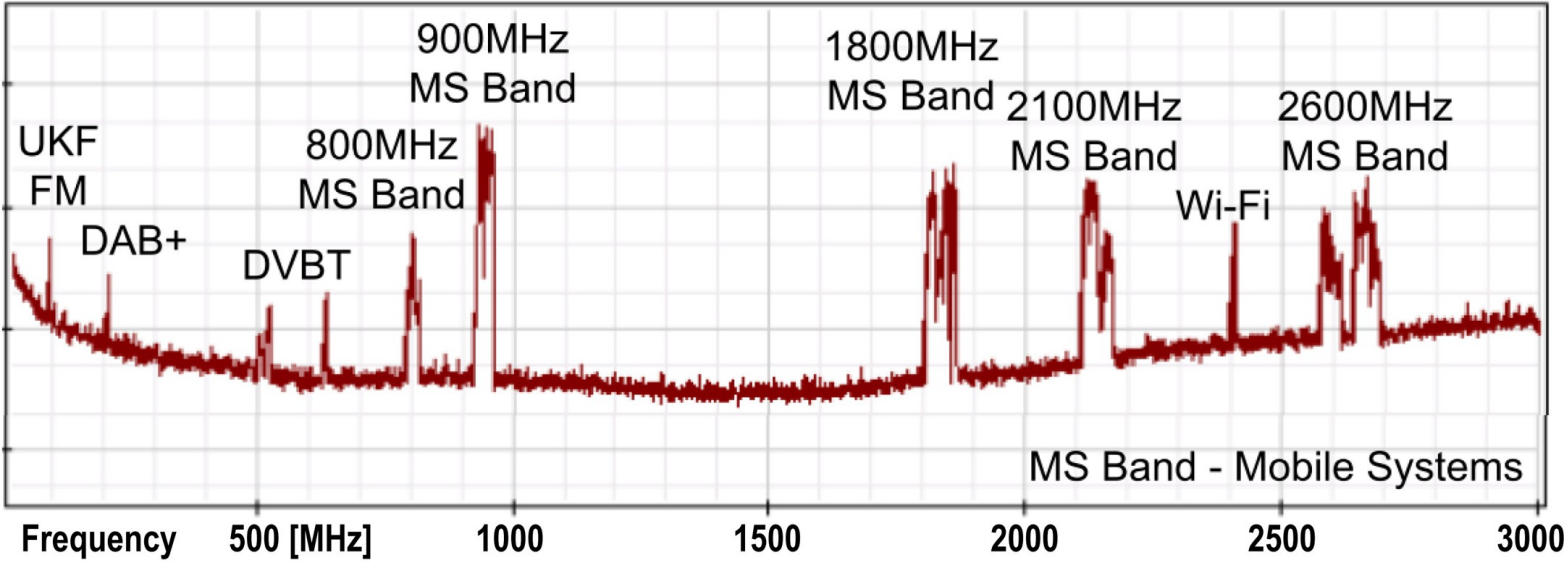
Electromagnetic spectrum used by major radiocommunication systems in Poland.

Electromagnetic spectrum from radio frequency (about 80 MHz) up to microwave band (about 6 GHz) is filled with many widely used wireless systems: among others, radio and television broadcasting (FM, DVB-T), wireless internet access (Wi-Fi) and mobile systems (GSM, LTE and 5G) [1,2]. As an example of typical electromagnetic field (EMF) spectrum occupation, Fig 1 and Table 1 present spectrum and band-plan in Poland. Some of those systems work continuously–with constant power level or in power dependent on current transmission mode. Most of the systems work using low-radiating power—continuously or transmitting in a specific time periods (with e.g. time-division multiplexing—TDM) and cannot be detected in every moment.

**Table 1:** Band assignment for mobile systems in Poland.

Band	Frequency spectrum	Typical use
700 MHz	694–790 MHz	5G soon
800 MHz	UL 791–821 MHz DL 832–862 MHz	LTE
900 MHz	UL 876–915 MHz DL 921–960 MHz	GSM, UMTS
1800 MHz	UL 1710–1785 MHz DL 1805–1880 MHz	GSM, LTE
2100 MHz	UL 1920–1980 MHz DL 2110–2170 MHz	LTE, UMTS
2600 MHz	2500–2690 MHz	LTE, 5G
3600 MHz	3400–3800 MHz	5G soon

UL–Up Link–transmission from the terminal to the base station, DL–Down Link–transmission from the base station to the terminal.

With constant development of information and communication technologies a trend towards achieving high transmission speed is observed. In mobile communication systems it is strictly connected with the occupation of unused high-frequency bands in order to grab wide bandwidth sectors. Simple explanation is delivered by Shannon-Hartley theorem which states that the channel capacity is a function of bandwidth and signal to noise ratio (Eq 1).

$$C[b/s]=B\cdot {\text{log}}_{2}\left(1+\frac{S}{N}\right)$$ (1)

where: C- channel capacity [bits/second], B–bandwidth [Hz], S/N—signal to noise

power ratio.

According to Eq 1 the simplest and most effective way to improve channel capacity is extending the channel bandwidth. Unfortunately, since the 20th century all bands below 1 GHz are tightly filled by communication systems and there is no possibility to implement a new one or extend existing one. Exploration and occupation of frequencies beyond 1 GHz has become a solution to overcome this limitation. However, this comes at a cost of higher signal attenuation (*L*) and is followed by the decrease of the transmission range, as described by the basic equation for free space attenuation of electromagnetic wave (2)

$$L[dB]=32,44+20log_{10}(f)+20log_{10}(d)$$ (2)

where: *L*–attenuation of electromagnetic (EM) wave in free space [dB], f–frequency [MHz], d–the distance between the transmitter and the receiver [km]

A compromise between transmission range and speed had been reached near 900 MHz band and this band is one of the most commonly used for radiocommunication systems, both in the rural areas (due to high transmission range) and in the city centers (due to better penetration of buildings than can be achieved using EMF of higher frequency) [1,2]. Simultaneously, communication systems became more scattered and based on many dense-deployed base-transceiver systems (BTS) working with low radiating power. This approach provides higher network capacity, especially needed in an urban area. Also, dense BTS deployment allows the transceiver antennas to be positioned relatively low (typically 15–50 m above ground) and covering the whole area with a much more uniform EMF distribution with relatively high power levels in the BTS neighborhood [3,4].

The clear upward trend in the presence of radio-frequency electromagnetic field (RF-EMF) and microwaves (MW) in the environment and the daily contact with wireless communication devices generating RF-EMF made it necessary to control radiated power levels and assess the level of exposure to a field with specific properties. Appropriate environment regulations for EM exposure levels set limits e.g. for public access places or workplace environments. Best known are the ICNIRP guidelines, but local regulations had also been established [5]. The electric field (E-field) strength limit in the RF-EMF and MW bands is frequency-dependent and typically remains in the range of 6 V/m to 61 V/m.

While these regulations were made with human well-being in mind, it is obvious that all living organisms can be affected by the EMF and among them–honey bees, the main representative of pollinators. Honey bees are essential for biodiversity and food production. While foraging they can be exposed to electromagnetic fields, especially in urban areas. Urban beekeeping has been a booming trend over the past few years [6] and since many electromagnetic emitters are localized in the cities we had decided to investigate their influence on honey bees.

So far, several observations of the negative impact of the low-frequency EMF (50 Hz) on the honey bees had been reported. It has been shown that the exposure to 50 Hz EMF caused weakening of the sense of smell, memory loss and reduction of cognitive skills [7]. Attention is also drawn to the decreased number of worker bees returning to the nest due to the problems with location recognition and general disorientation caused by the presence of the EMF. On the molecular level, the exposure of honey bees to 50 Hz EMF caused bee’s hyperactivity at the end of the season, coupled with the increased enzyme activities of AChE, CAT, GST, ALP involved in neural functionality, ROS defense, detoxifying activity and gut adsorption, respectively [8–11]. This in turn could cause problems with overwintering due to early stock exhaustion [12]. In line with these results it has been also shown that exposure to 50 Hz EMF caused reduction of glucose, triglycerides, and protein level in the hemolymph [13]. Also, this type of EMF caused decrease of the activity of AST and ALT enzymes and creatinine level, and increase of the albumin level in the hemolymph–these effects dependent both on time of the exposure and the field intensity [14].

There are also observations regarding the influence of RF-EMF on honey bees, describing the general impact of RF-EMF on the colony and/or behavior of individual bees, such as: reduction in the number of individuals in colonies, extended homing flight duration, decrease in breeding efficiency, changes in flight direction (movement of bees toward the areas affected by RF-EMF), increase in the intensity and frequency of sounds characteristic for those announcing the impending danger [15–17]. While some of the above mentioned impacts of the RF-EMF can stem from disorientation caused by errant stimulus perception, other may be a result of the possible impact of RF-EMF on metabolism and/or cell signaling [18,19]. In this work we describe the changes of the levels of some of the stress-related markers (enzyme activity, biochemical markers and gene expression) in honey bees exposed to varying intensity and duration of RF-EMF.

## Materials and methods

### Bees

Queens originating from the same mother-queen colony were inseminated with the semen of drones from the same father-queen colony. Ten randomly picked queens were placed in isolators with empty Dadant combs (435×300 mm) for egg-laying. Each queen was kept in a separate colony. After 20 days of brood development, the combs were transferred to an incubator (temp. 34.4°C ± 0.5°C, relative humidity of 70% ± 5%) for emerging of the worker bees without the presence of the adult bees. All combs were transported at the same time and placed in the same incubator. Food (honey and pollen) was provided *ad libitum*.

### Exposure to radio-frequency electromagnetic field

The 900 MHz band was selected for exposure to microwave fields. It is a commonly used band in mobile telephony systems and has a large share in the total EM energy emitted to the environment by mobile systems (GSM, LTE, UMTS) (Fig 1). Due to dense locations of EM-field sources and high propagation capabilities, signals from the examined band can be observed at great distances covering urban and rural areas. E-field strengths of 12V/m, 28V/m and 61V/m were assumed as the exposure level. Additionally, Specific Absorption Rate (SAR) was estimated by an analytical method in accordance with literature data [20,21] and verified by simulation using CST simulation tool. The determined SAR value was 0.05 W/kg at 12V/m, 0.3 W/kg at 28V/m and 1.4 W/kg at 61V/m. The uncertainty of determining the SAR is estimated at 30%. Furthermore, calculated SAR values are significantly lower than the values that cause significant thermal effect on honey bees.

Randomly picked one-day-old worker bees were placed in wooden cages (20 × 15 × 7 cm). Each cage contained 100 worker bees and two inner feeders with a 50% sucrose solution. Bees were fed *ad libitum*. Each experimental group consisted of 10 cages. The bees were randomly assigned to 9 experimental groups which were exposed to the selected EMF intensities for 15 min, 1 h and 3 h. The control group was not exposed to the RF-EMF.

**Fig 2:**
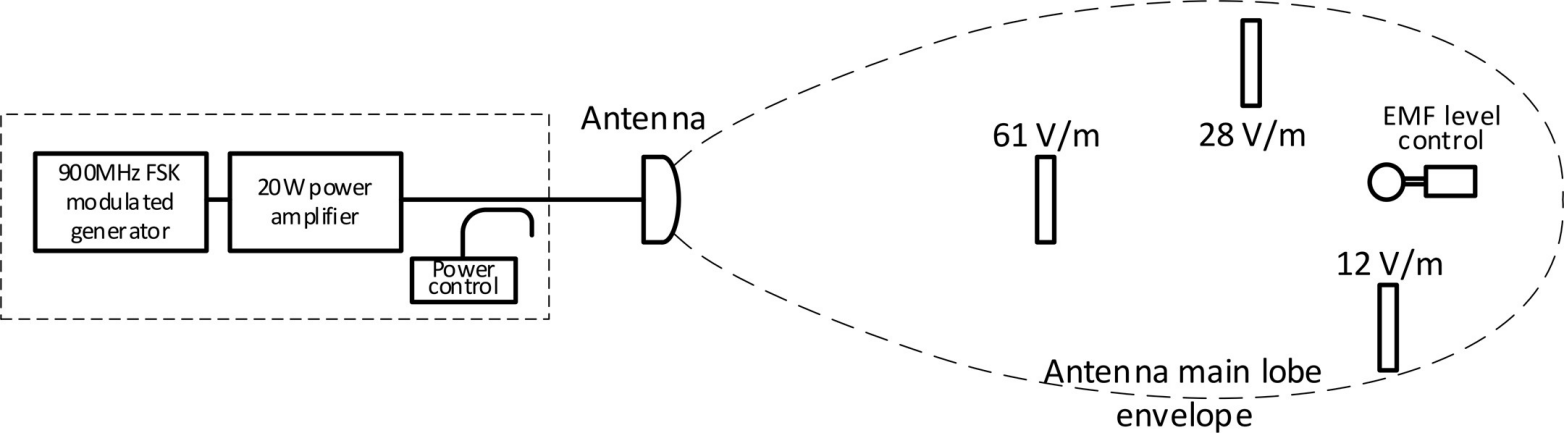
Exposure setup scheme with EMF level control system.

The RF-EMF source consists of a panel antenna connected to an amplifier powered by a radio frequency generator working at 900 MHz with narrowband FSK (Frequency Shift Keying) modulation. The RF-EMF exposed cages with bees were placed in the free space of the antenna’s far-field conditions—in the antenna’s main lobe area. Cages were arranged in a way preventing EM-coupling between the antenna and cages and cages themselves. A schematic of the system is shown in Fig 2. Cages with bees were positioned at three fixed points - 61V/m, 28 V/m, and 12V/m field intensity. The distribution of EMF in the testing area was measured using an NBM-520 S/N C-0062 meter with probe EF-1891 S/N A-0335 calibrated by an accredited calibration laboratory AP-078 (calibration certificate LWiMP/W/082/21). The measurements were carried out by the accredited testing laboratory LWiMP AB-361. EMF non-uniformity in the individual exposure areas did not exceed ±9% for 61 V/m area, ±7% for 28 V/m area and ±5% for 12 V/m area. Field strength variability throughout the measurement period did not exceed ±3% and was controlled by monitoring the power delivered to the antenna and controlling the field strength in the exposure area.

### Measurement of the enzyme activity and the level biochemical markers

One hundred bees was used for analysis of enzyme activity and the level of biochemical markers. The bees were randomly picked from each cage (10/cage) and the hemolymph was collected by removing the antennae of live bees using sterile tweezers [22].The samples were collected in 20 ul glass capillaries (end-to-end w/o anticoagulant). The capillaries were placed in 1.5 Eppendorf tubes filled with 150 ul milli-Q water; each tube contained 5 capillaries. The test tubes were cooled during the procedure and then transferred to -80°C.

#### AST, ALT and ALP

The reagent composition for AST, ALT and ALP was as following:

AST: 2-Ketoglutarate (13 mmol/L), L Aspartate (220 mmol/L), LDH (1200 U/L), MDH (90 U/L), NADH (10 mmol/L), Tris buffer (88 mmol/L) and EDTA (5.0 mmol/L), pH 8.1

ALT: 2-ketoglutarate (13 mmol/L), L-alanine (440 mmol/L), NADH (0.10 mmol/L), LDH (1800 U/L), Tris buffer (97 mmol/L) and EDTA (50 mmol/L), pH 7.8

ALP: 2-amino-2-methyl-1-propanol (900 mmol / L), magnesium acetate (1.6 mmol / L), zinc sulfate (0.4 mmol / L) and HEDTA (2, 0 mmol / L).

For AST or ALT activity measurement, 100 ul of appropriate reagent solution was mixed with 10 ul of the hemolymph, vortexed for 3–5 seconds and heated at 37˚C for 30 seconds. The absorbance was measured at four time points (0, 1, 2 and 3 minutes) after incubation at 340 nm.

For ALP activity measurement, 100 ul of the reagent was mixed with 2 ul of hemolymph, vortexed for 3–5 seconds and heated at 37˚C for 60 seconds. Then 20 ul of 4-NPP (16.0 mmol/L) was added and reaction solution was vortexed for 3–5 seconds and heated at 37˚C for 60 seconds. The absorbance was measured at four time points (0, 1, 2 and 3 minutes) after incubation at 405 nm.

The activities of AST, ALT and ALP were calculated according to the formulas:

$${\text{Activity}\;}_{\text{ALT}\;/\;\text{AST}\;/\;\text{ALP}}=\Delta \text{Abs}\;/\;\text{min}\times \text{F}$$

$${\text{F}}_{\text{ALT}\;/\;\text{AST}}\;=(\text{TV}\times 1000)/(6.3\times \text{SV}\times \text{P})$$

$${\text{F}}_{{}_{\text{ALP}}}=(\text{TV}\times 1000)/(18.8\times \text{SV}\times \text{P})\Delta \text{Abs}/\min=((\text{A}2-\text{A}1)+(\text{A}3-\text{A}2)+(\text{A}4-\text{A}3))/3$$

Where:

A1, A2, A3, A4—individual absorbance readings for samples

TV—total volume of the reaction mixture

SV—sample volume used for the reaction

P—length of the optical path of the cuvette

6.3—the absorption coefficient of dihydronicotinamide adenine dinucleotide (NADH; at a wavelength of 340 nm)

18.8—absorption coefficient for 2,4-dinitrophenol (2,4-DNP)

#### GGTP

GGTP activity was determined using ABX Pentra GGT CP assay kit (HORIBA ABX Diagnostics, France) according to the manufacturer’s recommendation. 10 ul of hemolymph was used in each analysis.

#### Biochemical markers

The level of biochemical markers was measured using dedicated kits from HORIBA ABX Diagnostics, France. Values in the parenthesis indicate the volume of hemolymph taken for given analysis. All analysis were performed according to manufacturer’s recommendations.

albumin: ABX Pentra Albumin CP (2 ul)

creatinine: ABX Pentra Enzymatic Creatinine CP (10 ul)

uric acid: ABX Pentra Uric Acid CP (5 ul)

urea: ABX Pentra Urea CP (3 ul)

### Gene expression analysis

For gene expression analysis, total RNA was extracted from the abdomens of the exposed bees (three bees/isolation) using ExtractMe Total RNA kit (Blirt) according to the manufacturer’s recommendation. Six micrograms of the RNA were treated with DNaseI (Turbo DNA-free Kit, Invitrogen) according to the manufacturer’s recommendation. One microgram of DNase-treated RNA was reverse-transcribed using Superscript IV enzyme (Invitrogen). Twenty microliters of the cDNA were diluted with 20 ul of water and 1 ul of the cDNA was used for a single Real-Time PCR reaction.

The Real-Time PCR reaction was performed using PowerUp SYBR Green MasterMix (Applied Biosystems) and QuantStudio3 apparatus (Applied Biosystems). The reaction conditions were identical for all analyzed genes and consisted of the following steps: 50°C for 2 min., 95°C for 2 min., and 40 cycles: 95°C for 15 sec., 60°C for 1 min.

The following primers were used:

rps5: forward 5’ - AATTATTTGGTCGCTGGAATTG, reverse 5’ - TAACGTCCAGCAGAATGTGGTA

ppo: forward 5’-AGATGGCATGCATTTGTTGA, reverse 5’—CCACGCTCGTCTTCTTTAGG

hsp10: forward 5’ - TGTTGTAGCAATTGGACCTGG, reverse 5’ - TGCCAGTATATCTGACTCACG

hsp70: forward 5’ - ATCAACCTGGCGTCTTGATTC, reverse 5’ - TGAGGTACACCTCTAGGTGC

hsp90: forward 5’ - GCGACAGAATGAAGTGCCATG, reverse 5’ - CAGAATCAAGTGGCAGGCTTG

vitellogenin: forward 5’ - TCAGTAACCAATGCGAGGGC, reverse 5’ - CGACATCTCGGTGTCCAATC

For each primer set the efficiency of the PCR reaction was identified using a series of 5-fold dilutions of one of the template cDNAs. The expression levels of the stress-related genes were normalized to the reference gene (rps5). All experiments were performed in triplicate. The gene expression levels identified in the honeybees exposed to RF-EMF are presented relative to the unexposed honeybees. Melting curve analysis was performed for each amplification product to verify the specificity of the reaction. Amplification of the DNaseI- treated RNA was performed for each sample to ensure that there was no genomic DNA contamination.

### Data analysis

We used to statistical analysis R 4.1.2 with RStudio (R Core Team 2021). In all tests, the level of significance was α = 0.05. Normality of data distribution was tested by Shapiro-Wilk test. Kruskal-Wallis test with Holm correction for multiple comparison was used to check differences between groups (package ‘agricolae’). Data was expressed as means ± standard deviation

## Results and discussion

**Fig 3:**
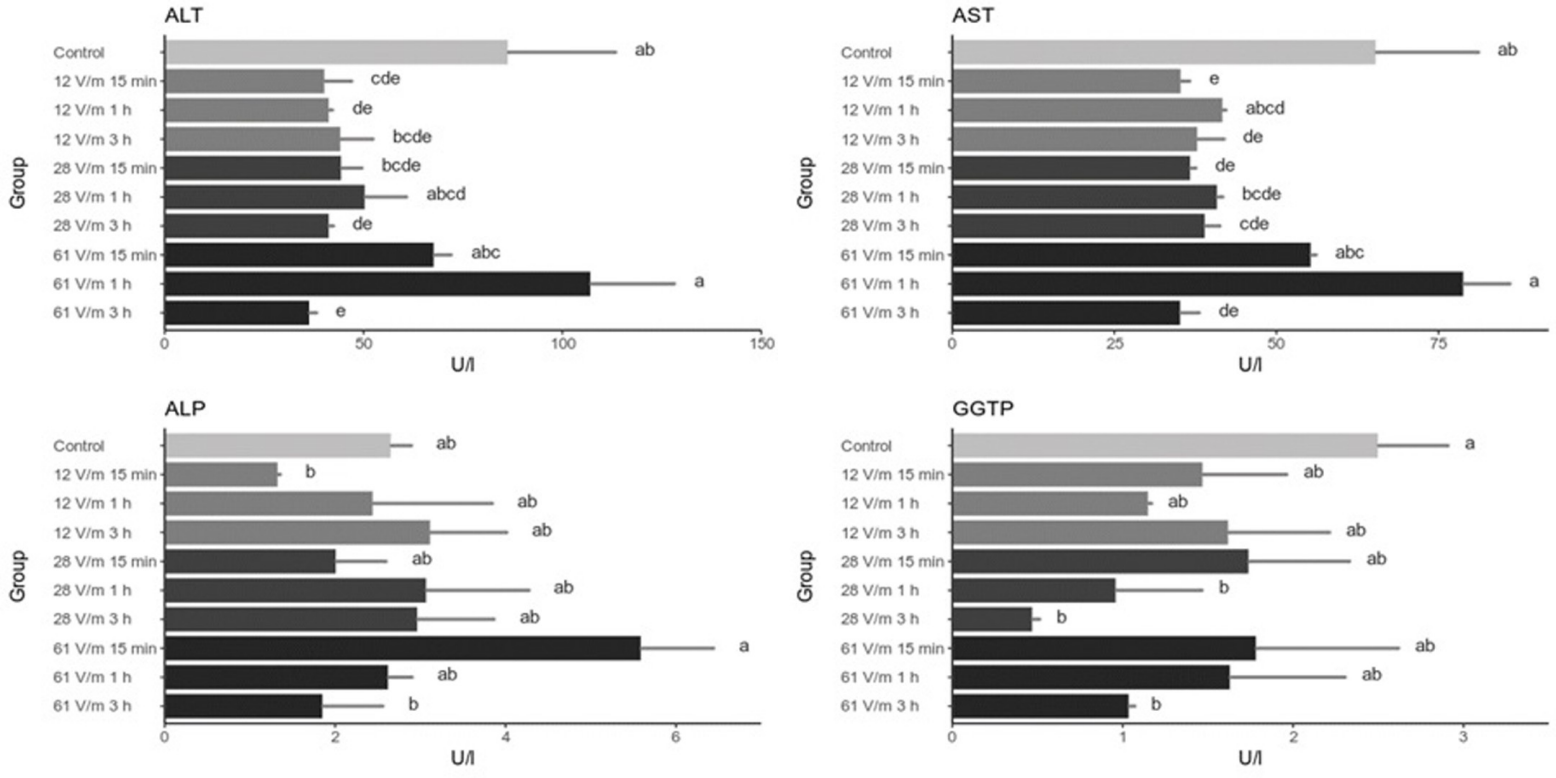
Activity of enzymes in the hemolyph of bees exposed to 900 MHz EMF. Bars represent mean values (n = 20) and the error bars the standard deviation The same letters within one graph indicate no statistically significant differences between the groups (Kruskal-Wallis test with Holm correction. α = 0.05).

In this work we have analyzed the changes of several biochemical and gene expression markers in response to the exposure of honey bees to 900 MHz RF-EMF in varying conditions (intensity and exposure duration). The markers have been chosen because they had been previously described as those implicated in stress-response in honey bees. Activities of four enzymes had been analyzed: alanine aminotransferase (ALT), aspartate aminotransferase (AST), alkaline phosphatase (ALP) and gamma-glutamyl transpeptidase (GGTP) (Fig 3). We show that the activities of ALT and AST generally decreased after the exposure to 900 MHz, in some cases significantly, however these changes were not correlated neither with duration of the exposure nor with the field intensity. The activity of ALP in the analyzed conditions has not changed significantly when compared to the control. A decrease (yet statistically not significant) in ALP activity was found in bees exposed to 12 V/m for 15 minutes and to 61 V/m for 3 hours. The activity of GGTP has dropped significantly in bees that were exposed to relatively high-intensity RF-EMF for long time periods.

**Fig 4:**
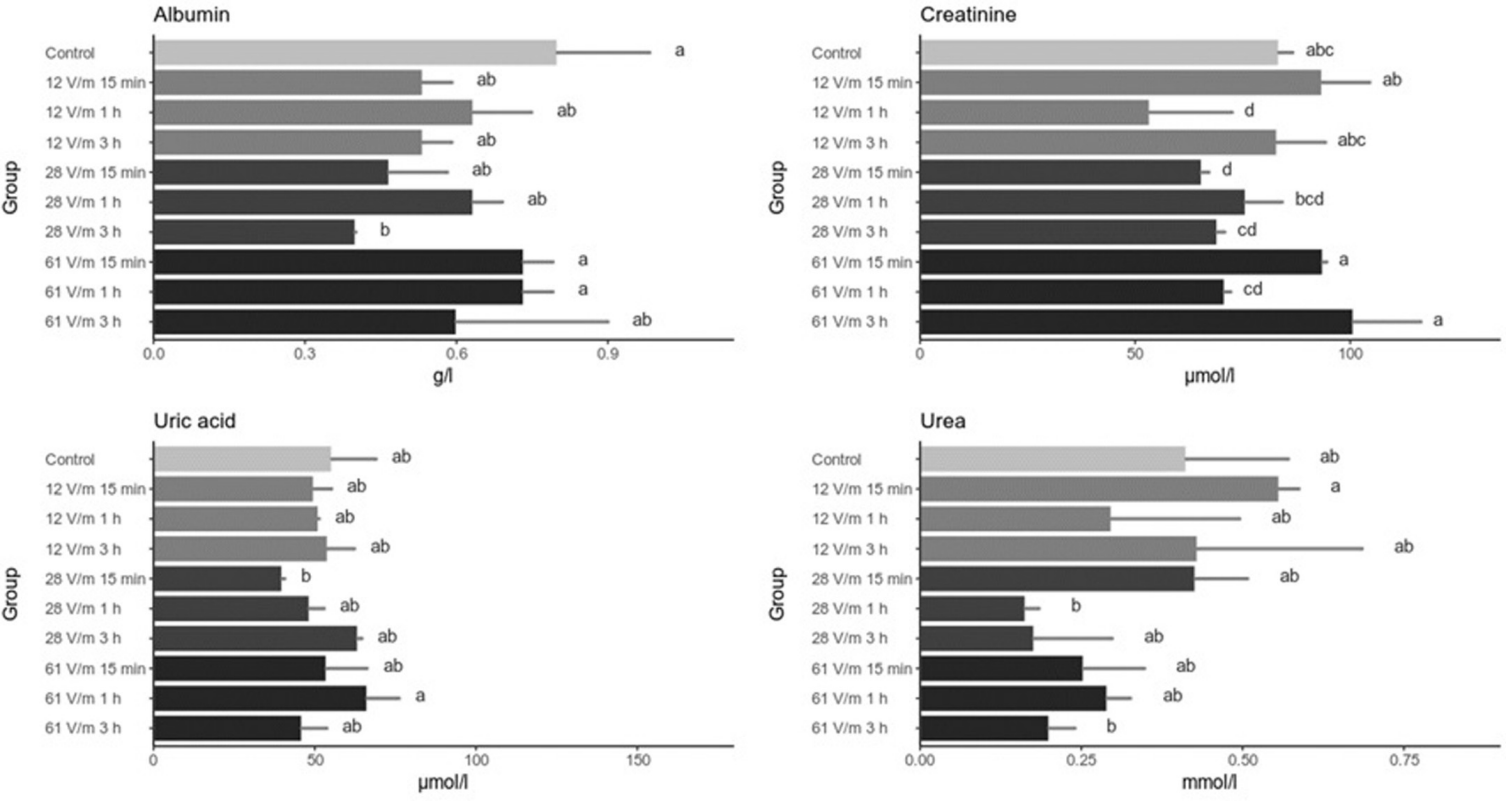
Level of biochemical markers in the hemolyph of bees exposed to 900 MHz EMF. Bars represent mean values (n = 20) and the error bars the standard deviation The same letters within one graph indicate no statistically significant differences between the groups (Kruskal-Wallis test with Holm correction. α = 0.05).

The levels of albumin and creatinine in the hemolymph generally did not change as a result of exposure to 900 MHz EMF (Fig 4). The only significant changes observed were the decrease of the albumin in the 28 V/m 3 hour group and creatinine in 12 V/m 1 hour and 28 V/m 15 minutes groups. The levels of uric acid had not changed significantly in any of the analyzed groups whereas a downward trend of the urea levels with increasing field intensity and exposure time can be observed (Fig 4).

**Fig 5:**
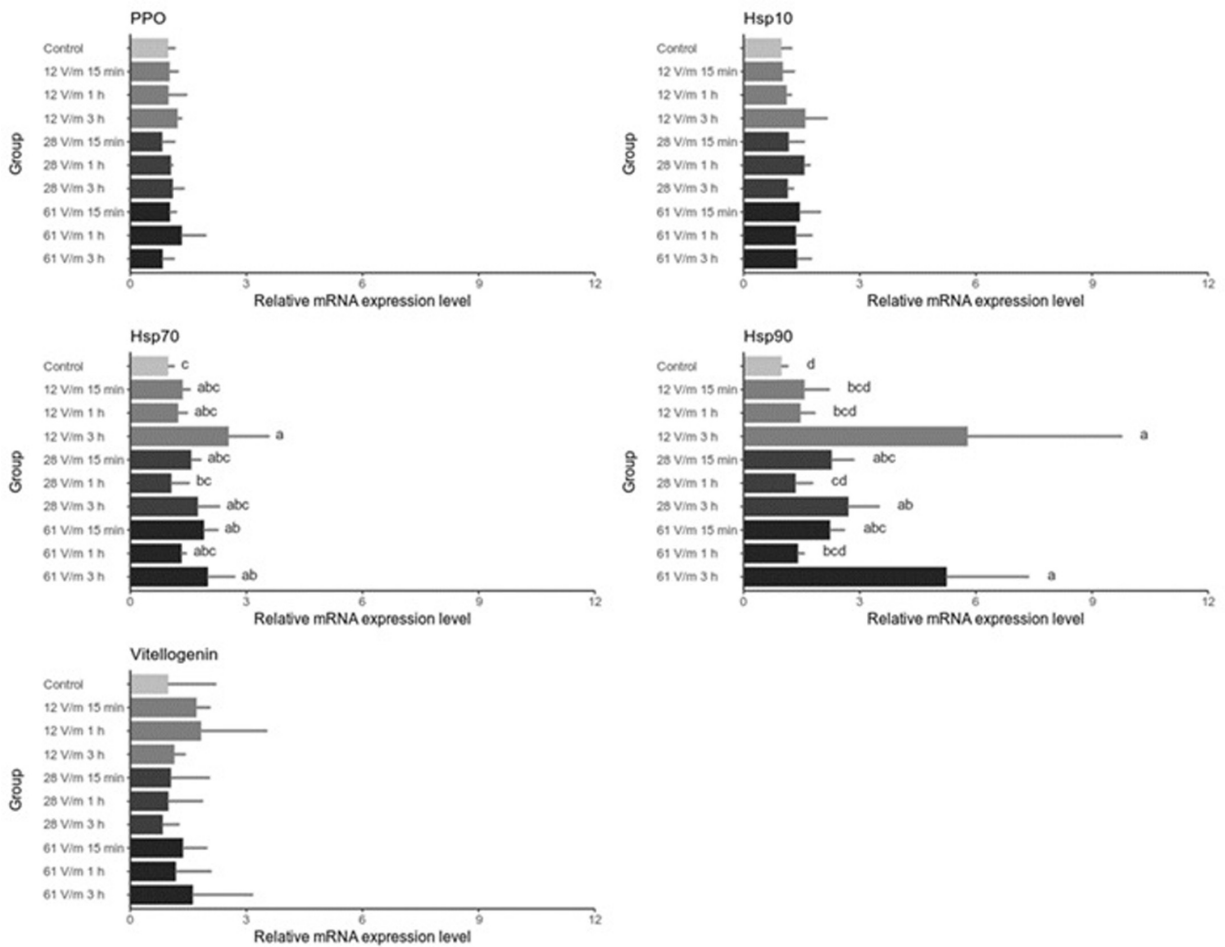
Relative expression of stress-related genes in abdomens of bees exposed to 900 MHz EMF. Bars represent mean values (n = 3) and the error bars the standard deviation The same letters within one graph indicate no statistically significant differences between the groups (Kruskal-Wallis test with Holm correction. α = 0.05).

In order to determine whether exposing bees to RF-EMF triggered stress response on the cellular level we have analysed by Real-Time PCR the expression profile of genes that were previously characterized as stress markers (Fig 5). Various stress factors, such as cold, heat, UV light and overcrowding were found to trigger heat shock response and upregulation of genes encoding the members of Hsp family [23]. Vitellogenin has also been suggested as a stress marker that has a protective role against oxidative stress. The expression of rps5 gene was used as endogenous control and results are normalized to expression levels identified in unexposed bees. We have also analyzed the expression level of ppo (polyphenol oxidase) gene as a control of gene unrelated to the analyzed factors. As expected, we have found no differences in ppo expression in the analyzed groups when compared to the control and between the analyzed groups. No differences were also found in hsp10 and vitellogenin genes expression. We have observed a statistically significant and relatively high upregulation of hsp90 gene, especially in groups exposed to RF-EFM for long time periods. It is also worth noticing that the expression pattern of hsp90 in the analysed groups is mirrored by the expression pattern of hsp70, although upregulation of hsp70 after the RF-EMF exposure is not as high as hsp90.

When compared to our previous studies involving exposing honey bees to 50 Hz EF, the effects of exposing bees to 900 MHz EMF on the levels of biochemical markers are markedly different. Firstly, 900 MHz caused decrease in ALT and AST only and no changes were detected in the levels of ALP, creatinine and albumin. After the exposure to 50 Hz the levels of ALP activity and creatinine decreased while albumin increased [11]. Secondly, the effects on the levels of ALT and AST after the exposure to 50 Hz EF were both time- and field intensity- dependent while no such correlations have been observed after the exposure to 900 MHz EMF. These differences indicate that it is warranted to investigate the impact of the exposure of bees to various ranges of electromagnetic spectrum and consider EMF of different frequency as distinct stress factors.

The decrease of ALT and AST activity could point to the influence of RF-EMF exposure on protein metabolism in which both enzymes play crucial role; it is worth noting that the changes of the activity of ALT and AST in the analyzed groups are correlated. It has been shown that various stress factors can cause the disruption of proteostasis–the balance between protein synthesis, folding and degradation on the level of the cell and in the whole organism [24]. Factors disturbing proteostasis lead to the build up of unfolded proteins that triggers cellular responses which eventually limit the damages and cause restoration of homeostasis. One of those mechanisms is Heat Shock Response (HSR) involving heat-shock proteins (HSPs) [25]. The upregulation of HSPs have been previously described in honey bees under different stress conditions; here we show that bees exposed to RF-EMF upregulate Hsp70 and Hsp90 expression. At first glance one may conclude that this may be the evidence of heat stress caused by RF-EMF, however we draw different conclusions. As shown in previous studies, increased temperature, apart from Hsp70 and Hsp90, also caused upregulation of Hsp10 and vitellogenin gene expression [23,26] which in our study remain unaffected. It has been also reported that heat shock results in higher activity of ALT, AST, ALP and GGTP enzymes whereas in our study we have observed generally lower activity of ALT, AST, GGTP and unaffected activity of ALP in bees exposed to RF-EMF. In terms of changes in the stress-related gene expression, the exposition of bees to RF-EMF is similar to the reported previously changes caused by UVB irradiation i.e. increased expression of Hsp70 and Hsp90 genes with no changes in Hsp10 and vitellogenin expression [23]. This may suggest that similarly to UVB exposition, RF-EMF may cause the induction of Hsp70 and Hsp90 in order to protect or rescue damaged proteins. On the other hand, lack of significant changes in vitellogenin gene expression, may suggest that RF-EMF does not cause (at least immediate) changes in the metabolic rate, yet again indicating distinct effects caused by 50 Hz EF and RF-EMF.

To summarize, our study shows that exposure to 900 MHz electromagnetic field induces response of the honey bees that can be detected on the level of enzyme activity and the expression of stress-related genes. The response is similar to the one previously described as a result of exposition to UVB irradiation and most likely cannot be attributed to increased temperature. The experiment was performed in the 900 MHz band, representing the lower frequency bands used in cellular telephony systems. Further research work is planned using higher frequencies. While it was necessary to conduct laboratory research first, broadening the research to field studies should provide more detailed information about RF-EMF impact on honey bees as individuals may differ in their sensitivity and degree of exposure to RF-EMF based on their sex, age, and role which they play in the colony.
